# Kasabach–Merritt Syndrome, an underdiagnosed swollen leg in newborn with anemia and jaundice

**DOI:** 10.1002/ccr3.8089

**Published:** 2023-11-01

**Authors:** Chariya Chap, Sakviseth Bin

**Affiliations:** ^1^ Neonatal Ward National Pediatric Hospital Phnom Penh Cambodia; ^2^ Neonatal Intensive Care Unit Calmette Hospital Phnom Penh Cambodia

**Keywords:** jaundice, Kasabach–Merritt syndrome, neonatal anemia

## Abstract

**Key Clinical Message:**

Kasabach–Merritt syndrome is a rare disease. Early recognition, timely transfer, and proper management are crucial in reducing bleeding complication and mortality.

**Abstract:**

The authors report a rare case of Kasabach–Merritt syndrome in a Cambodian neonate presenting with anemia and jaundice at Day 5 of life. Since the mass was not recognized at birth, the diagnosis and treatment were delayed, thus leading to demise.

A 5‐day‐old boy was brought by his parents to our neonatal unit for jaundice and reduced feeding. The initial physical examination revealed jaundice (transcutaneous bilirubin 20 mg/dL), severe anemia, and, unexpectedly, the swollen and cyanotic right thigh, which was, according to the parents, noted since birth with increasing size (Figure 1). Kasabach–Merritt syndrome (KMS) was suspected and confirmed by ultrasound findings (homogeneous hyperechoic mass extending from the thigh to calf), thrombocytopenia (platelets 55 × 10^9^/L), and hypofibrinogenemia (fibrinogen 1.5 g/L). Cranial and abdominal ultrasound at admission were unremarkable. The infant was referred to intensive care unit (ICU) where he was treated with intensive and continuous phototherapy while receiving one pack of red blood cells and one pack of platelets. However, in spite of the urgent symptomatic managements, the boy succumbed 12 h after the admission due to pulmonary hemorrhage.

**FIGURE 1:**
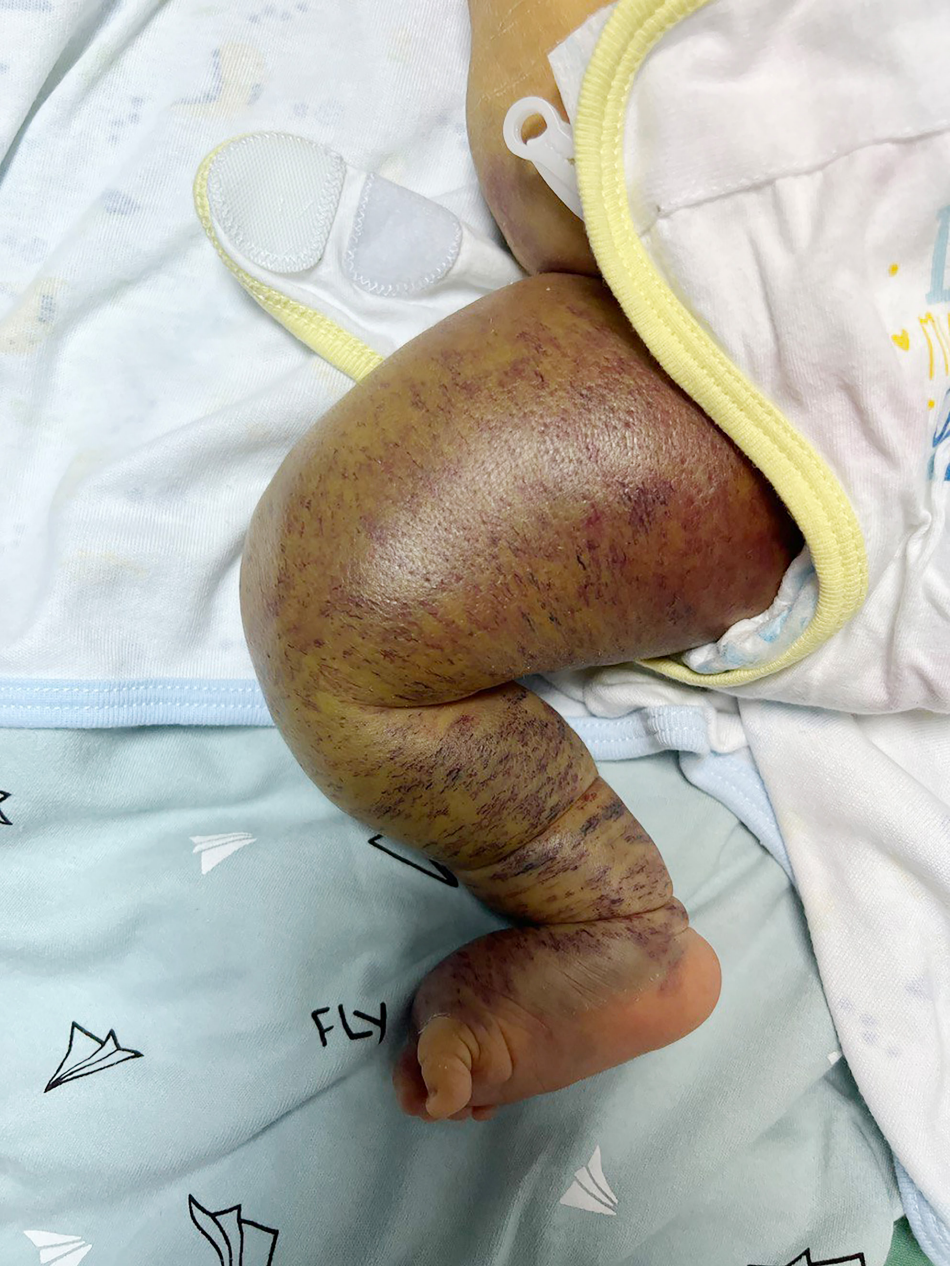
Swollen, renitent, cyanotic right thigh.

The term “Kasabach–Merritt syndrome” was coined after the first report of a newborn with a distinctive association between a rapidly extending hemangioma and extensive purpura in 1940.^1^ Later, retrospective studies demonstrated that KMS was not linked to simple congenital or infantile hemangioma, but it was the complication of two specific capillary hemangioma: Kaposiform hemangioendothelioma (KHE) and tufted angioma.^2^ Biopsy of the lesions can confirm and differentiate the entities, yet its indication is limited due to the risk of bleeding. Imaging studies (ultrasound and MRI) are more commonly useful. The curative medical treatments rely mostly on corticosteroids, alpha interferon, and vincristine.^3^ The mortality rate was about 24%.^2^

Our case demonstrated a typical KHE, complicated with thrombocytopenia and coagulopathy. Multi‐disciplinary approach was sought. However, due to late recognition and transfer, biopsy and surgery could not be done, and steroids were also suspended due to bleeding risks. Vincristine was not available in our center.

## AUTHOR CONTRIBUTIONS

**Chariya Chap:** Investigation; writing – original draft; writing – review and editing. **Sakviseth Bin:** Conceptualization; supervision; validation; writing – original draft; writing – review and editing.

## FUNDING INFORMATION

There was no funding support for the case report.

## ETHICS STATEMENT

No ethical approval is required.

## CONSENT STATEMENT

Written informed consent was obtained from the patient's parents and is available for review upon request.

## Data Availability

The data that support the findings of this study are available from the corresponding author upon reasonable request.

## References

[1] Kasabach HH , Merritt KK . Capillary hemangioma with extensive purpura: report of a case. Am J Dis Child. 1940;59(5):1063‐1070.

[2] Sarkar M , Mulliken JB , Kozakewich HP , Robertson RL , Burrows PE . Thrombocytopenic coagulopathy (Kasabach–Merritt phenomenon) is associated with Kaposiform hemangioendothelioma and not with common infantile hemangioma. Plast Reconstr Surg. 1997;100(6):1377‐1386. 9385948 10.1097/00006534-199711000-00001

[3] Kelly M . Kasabach–Merritt phenomenon. Pediatric Clin North Am. 2010;57(5):1085‐1089. 10.1016/j.pcl.2010.07.006 20888459

